# β-Glucan–mediated macrophage priming enhances macrophage activation and host defense against *Mycobacterium tuberculosis* infection

**DOI:** 10.3389/fmicb.2026.1952695

**Published:** 2026-10-09

**Authors:** Firoz Ahmad, Mohd Khubaib, Rolee Sharma

**Affiliations:** 1 Department of Physiological Sciences, Oklahoma Centre for Respiratory and Infectious Diseases, Oklahoma State University, Stillwater, OK, United States; 2 Department of Biosciences, Integral University, Lucknow, Uttar Pradesh, India; 3 Department of Life Sciences & Biotechnology, CSJM University, Kanpur, Uttar Pradesh, India

**Keywords:** host-directed therapy, macrophage, phagolysosome, priming, β-glucans

## Abstract

Tuberculosis remains a major cause of infectious mortality worldwide, highlighting the need for complementary therapeutic strategies beyond conventional antibiotics. Host-directed approaches may enhance macrophage-mediated control of infection. In this study, we investigated the effects of β-glucan priming combined with drug-loaded yeast-derived β-glucan particles (DYDGP) in an *in vitro* macrophage model of *Mycobacterium tuberculosis* infection. Phorbol 12-myristate 13-acetate (PMA)-differentiated THP-1 macrophages were compared across unprimed, β-glucan-only, antibiotic-only, and β-glucan-primed/DYDGP-treated groups. β-Glucan priming combined with DYDGP treatment was associated with enhanced receptor-mediated phagocytic activity, improved intracellular bacterial control, and preserved macrophage integrity. These effects were associated with increased intracellular reactive oxygen species (ROS) production, enhanced phagolysosomal maturation, and altered autophagic activity. Colony-forming unit (CFU) analysis showed that β-glucan-primed macrophages treated with DYDGP exhibited a significantly lower bacterial burden than the unprimed, β-glucan-only, and antibiotic-only groups at 1, 6, and 24 h post-infection. Unprimed macrophages showed progressive bacterial growth, with log CFU values of 4.2, 4.8, and 5.2, whereas β-glucan-primed/DYDGP-treated macrophages showed reduced values of 3.3, 2.5, and 2.1, respectively. β-Glucan-only treatment resulted in log CFU values of 4.2, 4.4, and 4.7, while antibiotic-only treatment resulted in values of 4.2, 4.1, and 3.9 at the corresponding time points. These findings suggest that β-glucan priming combined with DYDGP treatment may enhance intracellular control of *M. tuberculosis* in this macrophage model and support further investigation of this approach as a potential adjunctive strategy for tuberculosis.

## Graphical abstract

**Figure:**
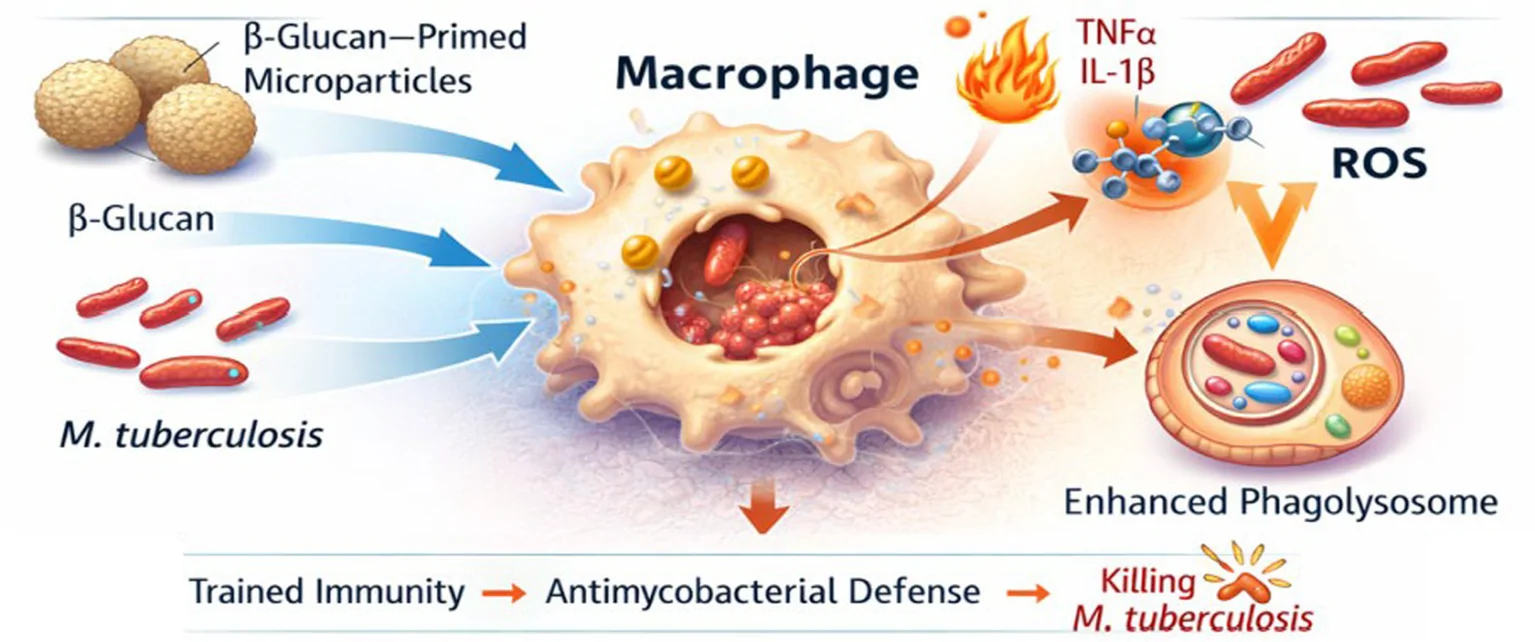

## Introduction

*Mycobacterium tuberculosis*, the causative agent of tuberculosis (TB), remains a major global health burden and is responsible for millions of deaths annually. A hallmark of *M. tuberculosis* infection is its ability to persist within host macrophages and survive for extended periods within granulomas by subverting immune responses (Bagcchi, 2023). This persistence underscores the need for innovative strategies that go beyond conventional antimicrobial therapies. Host-directed therapies have emerged as a promising approach to enhance immune-mediated control of infection. Among these, β-glucans—naturally occurring polysaccharides found in microbial cell walls—have gained attention because of their potent immunomodulatory properties. β-Glucans are recognized as pathogen-associated molecular patterns (PAMPs) and interact with pattern-recognition receptors such as dectin-1, leading to activation of innate immune responses and modulation of inflammatory signaling pathways (Saeed et al., 2014; Soto et al., 2012; Ahmad et al., 2024a,b,c; Pan et al., 2020; Verrall et al., 2020; Fatima et al., 2021; Quintin et al., 2012). These properties have been widely explored in biomedical applications, including their use as biocompatible carriers in drug-delivery systems approved by regulatory agencies such as the United States Food and Drug Administration (USFDA) and the European Food Safety Authority (EFSA) (Sirgel et al., 2013; Ahmad et al., 2024a,b,c; Cohen et al., 2016; Tesz et al., 2011).

Recent studies have highlighted the ability of β-glucans to induce long-lasting functional changes in innate immune cells through metabolic and epigenetic reprogramming, a phenomenon often referred to as primed immunity. However, depending on the experimental design, β-glucan exposure may also result in short-term immune priming that enhances antimicrobial responses without a defined resting phase. In this context, macrophage activation through β-glucan-mediated macrophage priming has been shown to enhance cytokine production, oxidative stress responses, and intracellular pathogen clearance (Netea et al., 2016; Braian et al., 2023; Joosten et al., 2018; Pan et al., 2020, 2022; Betz et al., 2011).

Building on these insights, we investigated the immunomodulatory potential of β-glucans using drug-loaded yeast-derived β-glucan particles (DYDGP), a microparticle-based system designed to combine immune activation with targeted drug delivery. This dual-function platform enables simultaneous modulation of host immune responses and intracellular delivery of antimycobacterial agents. Our previous work demonstrated that such systems enhance macrophage antimicrobial activity and promote oxidative stress-mediated killing of *M. tuberculosis* (Pan et al., 2020; Verrall et al., 2020; Fatima et al., 2021; Quintin et al., 2012). In the present study, we aimed to evaluate whether β-glucan-based priming, delivered through DYDGP, can enhance macrophage functional responses and improve intracellular control of *M. tuberculosis.* By integrating immunomodulation with targeted drug delivery, this approach seeks to advance host-directed therapeutic strategies and provide new insights into immune-based interventions for tuberculosis.

## Materials and methods

### Synthesis and formulation of rifabutin-loaded β-glucan microparticles

Synthesis and formulation of rifabutin-loaded inhalable β-glucan particles were performed using the alkaline-acidic extraction (AAE) method with dry baker’s yeast (*Saccharomyces cerevisiae*). Baker’s yeast was heated for 40 min, centrifuged, and washed; the semisolid mate rial was treated with acetone and isopropyl alcohol and sonicated to disperse the aggregated particles. The material was then spray-dried to obtain glucan particles. For drug loading, the particles were incubated with rifabutin, and sodium alginate was then added dropwise to seal their pores, as described previously (Ahmad et al., 2024a,b,c). See Supplementary Data S1 and Table 1.

**Table 1:** Spray-drying parameters and characteristics of blank YDGP β-glucan particles.

Batches	Inlet temperature (°C)	Aspirator	Outlet temperature (°C)	Pump flow rate (mL/min)	Yield (g)	Yield (%)	Drug-to-polymer ratio (g:g)	Leucine concentration (mg/g)
YDGP-A	145 °C	50%	45 °C	5 mL/min	3.48 g	17.5	1:1	100 mg
YDGP-B	150 °C	50%	50 °C	5 mL/min	4.08 g	20.5	1:1	200 mg
YDGP-C	155 °C	50%	55 °C	5 mL/min	4.48 g	22.5	1:1	300 mg

Characteristics of drug-loaded glucan particles (GP)						
Batches	*d* 0.1 (μm)	*d* 0.5 (μm)	*d* 0.9 (μm)	Drug-loading efficiency (%)	Mass median aerodynamic diameter (MMAD; μm)	Geometric standard deviation (GSD)
DYDGP-A	0.210 μm	5.800 μm	19.315 μm	67%	1.58	1.43
DYDGP-B	0.374	4.320	6.341	68%	2.07	1.81
DYDGP-C	0.908 μm	1.413 μm	3.496 μm	70%	1.02	1.63

### Human macrophage cell culture

For the host–pathogen interaction studies, the human macrophage THP-1 cell line was procured from the National Centre for Cell Science (NCCS), Pune, and cultured in RPMI 1640 medium (Sigma) with 10% fetal bovine serum (FBS; Gibco) and 1% antibiotic–antimycotic solution. THP-1 cells were treated with 50 ng/mL phorbol 12-myristate 13-acetate (PMA; Sigma) for 24 h to induce adherence, washed with incomplete medium, and maintained in complete medium for another 24 h. Cell-culture flasks and well plates were purchased from Eppendorf. Diphenylodonium (DPI; 10 μM), rotenone (100 μM), α-tocopherol (100 μM), rapamycin, and 7H11 agar medium were purchased from Sigma. Dimethyl sulfoxide (DMSO) was purchased from Merck, and all solvents were cell-culture grade.

### *Mycobacterium tuberculosis H37Rv* culture

For the mycobacterial infection studies, the *H37Rv* strain was obtained from Dr. Amita Jain, Head of the Department, Microbiology Division, King George Medical University (KGMU), Lucknow, India. Mycobacterial stocks were propagated in Middlebrook 7H9 medium (Difco), prepared in low-endotoxin water (Sigma) and supplemented with albumin–dextrose–catalase supplement (Becton Dickinson), 0.05% Tween 80 (Difco), and 0.5% glycerol (Sigma). Aliquots were stored at −80 °C, thawed, and propagated in Middlebrook 7H9 medium to the mid-exponential (log) phase at 37 °C. All experiments related to macrophage infection biology were performed in a biosafety level 3 (BSL-3) facility at the Department of Microbiology, KGMU, Lucknow, India.

### *In vitro* human macrophage cell priming

For *in vitro* priming, we seeded cells at 1 × 10^5^ cells per well in 96-well plates to allow adherence. The adherent human macrophage cells (THP-1) were then treated with 10 μg/mL β-glucans for 1, 6, or 24 h. The cells were then washed with warm, complete RPMI 1640 medium and incubated for 24 h before *M. tuberculosis* infection and treatment with 10 μg/mL DYDGP microparticles. We then analyzed the intracellular immune response generated by β-glucan-mediated macrophage priming and treatment in infected human macrophages. The response was visualized using a fluorescence microscope at ×100 magnification.

### *In vivo* priming followed by *ex vivo* alveolar macrophage harvest

For *in vivo* β-glucan priming experiments, 6-week-old male BALB/c mice were used. Animals were maintained under standard laboratory housing conditions with controlled temperature, humidity, and a regular light/dark cycle, with free access to food and water. The total number of animals used in each experimental group was *n* = 6, and animals were randomly allocated to the respective treatment groups. All animal procedures were performed in accordance with the guidelines of the Institutional Animal Ethics Committee of Chhatrapati Shahu Ji Maharaj University, Kanpur (IAEC approval no. IAEC/UIP/August2023/025, dated 12.08.2023). Mice were used solely for *in vivo* β-glucan exposure, followed by alveolar macrophage isolation and ex vivo phagocytic assays; no *in vivo M. tuberculosis* infection or therapeutic experiments were performed. Mice were treated with 10 μg/mL β-glucans at various time points (1, 6, and 24 h) through the nasal inhalation route for the defined period. After the priming period, mice were euthanized using 5% isoflurane in oxygen until loss of consciousness and cessation of respiration. Animals were maintained under isoflurane exposure for an additional 1–2 min, and death was confirmed by cervical dislocation before bronchoalveolar lavage fluid (BALF) collection for the isolation of alveolar macrophages (AMs), following previously described methods (Ahmad et al., 2024c). The collected lavage was centrifuged at 450 × *g* for 10 min, and the supernatant was discarded. The resulting alveolar macrophages were transferred to 6-well plates and cultured in RPMI 1640 medium supplemented with FBS and antibiotic/antimycotic. After incubation for 24 h, the cells were treated with fluorescently tagged particles to analyze receptor-mediated phagocytic uptake by alveolar macrophages. Cells that internalized microparticles displayed bright red fluorescence and were visualized using a fluorescence microscope at ×100 magnification.

### Human macrophage cell infection

Before infection, cells were seeded at a density of 1 × 10^5^ cells per well in 96-well plates (for quantitative studies) and 5 × 10^5^ cells per well in 6-well plates (for imaging and antimycobacterial studies). The adherent cells were first gently washed three times with 1–3 mL of incomplete medium without antibiotics. For cell infection, 3 mL of a log-phase *M. tuberculosis* culture was pelleted at 2,400 × *g* for 10 min, resuspended in 3 mL of culture medium, and passed seven to eight times through a 25-gauge syringe to obtain a single-cell bacterial suspension. The suspension was then transferred to a cuvette, and the optical density (OD) was measured at 600 nm to enumerate colony-forming units (CFU). A multiplicity of infection (MOI) of 5:1 (bacteria to host cells), calculated based on the measured optical density, was used for cell-infection experiments. *M.* tuberculosis-infected plates were incubated at 37 °C in 5% CO₂ for 1 h and subsequently washed three times with warm (37 °C) 1 × phosphate-buffered saline (PBS) to remove extracellular bacteria.

### Evaluation of the impact of priming on *Mycobacterium tuberculosis-*infected human macrophages

To assess the impact of priming on *M. tuberculosis*-infected human macrophages, 0.5 × 10^6^ cells were seeded in 6-well plates and treated with PMA for 24 h to induce adherence. After adherence, the cells were washed with medium and incubated again for 24 h. The cells were then infected with the H37Rv strain and assessed at 1, 6, and 24 h with or without DYDGP treatment. Cells were visualized using a fluorescence microscope at ×100 magnification.

### Assessment of the impact of priming on *in vitro* phagocytic uptake

To assess the impact of priming on *in vitro* phagocytic uptake, human macrophages were seeded at 1 × 10^6^ cells per well in 24-well plates and treated with PMA for 24 h to induce adherence. After adherence, the cells were washed with medium and incubated again for 24 h. The cells were then incubated with fluorescently tagged particles for 1, 6, or 24 h to assess particle uptake. The cells were washed three times with 1× PBS and visualized using an EVOS cell-imaging station at ×100 magnification.

### Evaluation of the impact of priming on reactive oxygen species generation in H37Rv-infected macrophages

*Mycobacterium tuberculosis*-infected macrophages were seeded at a density of 2 × 10^4^ cells per well in 96-well plates, loaded with 200 μL of 25 μM working 2′,7′-dichlorodihydrofluorescein diacetate (DCFH-DA) solution (diluted in RPMI), and incubated at 37 °C in 5% CO₂ for 30 min in the dark. The cells were then exposed to DYDGP microparticles (10 μg/mL) suspended in RPMI 1640 medium and incubated for 1, 6, or 24 h. Total fluorescence, indicating intracellular reactive oxygen species (ROS) production, was measured using an EVOS cell-imaging station at ×100 magnification.

### Evaluation of the impact of priming on acidic vesicular organelle formation in H37Rv-infected macrophages

To evaluate the impact of priming on acidic vesicular organelle (AVO) formation, acridine orange (AO) staining was used. AO, a weak base, permeates biological membranes in its uncharged form. Upon protonation, it selectively accumulates in AVOs, resulting in aggregates that exhibit intense red fluorescence. Cells exposed to DYDGP microparticles (MPs) were incubated with AO (1 μg/mL) for 15 min and assessed at 1, 6, and 24 h to facilitate AVO detection. The acidic compartments displayed pronounced red fluorescence (>650 nm), with fluorescence intensity correlating with the degree of acidity. Conversely, the nuclei and cytoplasm emitted green fluorescence (520 nm) upon excitation at the same wavelength. Imaging was conducted using an EVOS cell-imaging station at ×100 magnification.

### Assessment of priming impact on phagolysosomal fusion in *M. tuberculosis*-infected macrophages

To assess the impact of priming on *M. tuberculosis*-infected human macrophages, cells were first incubated with DYDGP microparticles (MPs) for 1, 6, or 24 h. Following incubation, the cells were washed with phosphate-buffered saline (PBS) to remove any unbound particles. To visualize acidic compartments, the cells were then stained with 50 nM LysoTracker-G. After incubation with the stain for 15 min at 37 °C, the cells were again washed with PBS to eliminate excess dye. The stained cells were subsequently examined using a fluorescence microscope at ×100 magnification. For quantitative analysis, relative fluorescence intensity was measured using ImageJ software to evaluate phagolysosomal fusion dynamics.

### Assessment of the impact of priming on intracellular growth of *Mycobacterium tuberculosis* in human macrophages

To evaluate the therapeutic effects of priming on host immunity, human macrophages were initially subjected to β-glucan priming or left unprimed for 1, 6, or 24 h and then infected with *M. tuberculosis.* The cells were subsequently treated with 10 μg/mL DYDGP, β-glucans, or antibiotic alone at the same particle concentration for 1, 6, or 24 h and then washed with PBS. The supernatant was discarded from infected and exposed macrophages, and 100 μL of sterile lysis buffer containing 0.05% (w/v) sodium dodecyl sulfate (SDS) in H₂O was added to each well. Serial dilutions of 100 μL of the lysates were then plated on 7H11 agar plates and incubated at 37 °C. Colonies were examined and counted after 3 weeks of incubation. Results are presented as the mean colony-forming unit count ± standard deviation (SD).

### Statistical analysis

All experiments were performed using independent biological replicates. Relative fluorescence intensity was quantitatively measured using ImageJ software. Statistical analyses were performed using Microsoft Excel. Comparisons among multiple experimental groups were performed using one-way analysis of variance (ANOVA), followed by Dunnett’s multiple-comparison *post hoc* test, with the designated control group as the reference. Dunnett’s test was used to account for multiple comparisons with the control. Data are presented as mean ± SD. All statistical tests were two-tailed, and *p* ≤ 0.05 was considered statistically significant.

## Results

### Priming enhances cellular morphology of macrophage cells

Our results highlighted significant differences in the morphological integrity of primed and unprimed macrophages treated with DYDGP particles. In primed, DYDGP-treated macrophages, the red arrows indicate that the membrane remained intact, exhibiting a well-defined structure with minimal disruption. The cytoplasmic content was preserved, indicating effective cellular function and resilience against the effects of *M. tuberculosis* H37Rv infection (Figure 1A). These cells showed no signs of blebbing or membrane rupture, suggesting that the priming process enhanced their ability to withstand the pathogenic assault. Conversely, unprimed, infected macrophages exhibited severe morphological changes. These cells showed significant membrane blebbing, a hallmark of cellular distress, and experienced marked loss of cytoplasmic content, as shown by the red arrows. This disruption was directly associated with *M. tuberculosis* infection, indicating compromised cellular integrity and function (Figure 1A).

**Figure 1:**
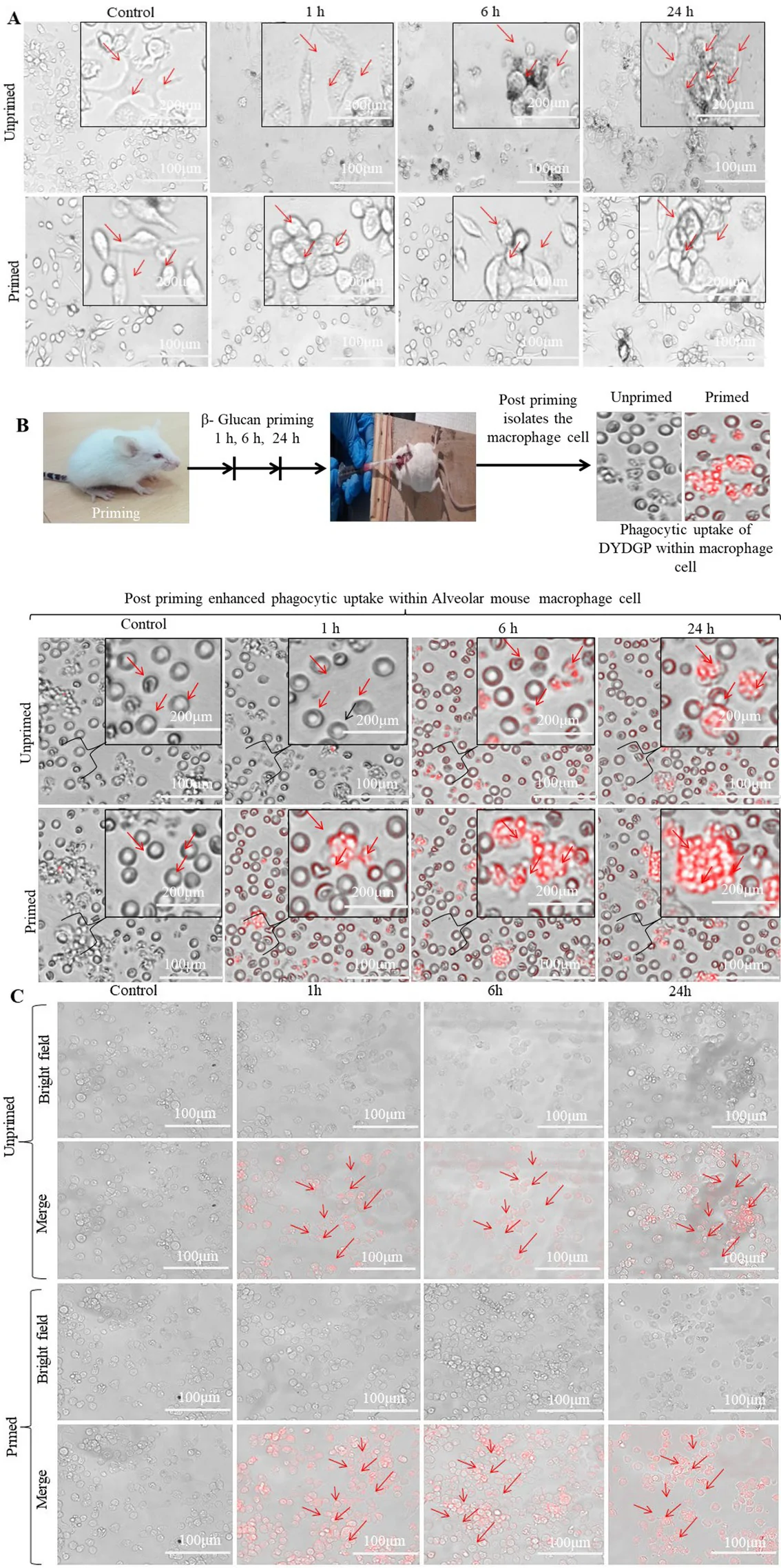
**(A)** Analysis of morphological changes in macrophages after *Mycobacterium tuberculosis* infection. The upper panel shows the unprimed control at 1, 6, and 24 h. The lower panel shows the primed control at 1, 6, and 24 h at ×100 and ×200 magnification. **(B)** Assessment of the phagocytic activity of alveolar macrophages: *in vivo* uptake of particles. The upper panel shows the unprimed control at 1, 6, and 24 h. The lower panel shows the primed control at 1, 6, and 24 h at ×100 and ×200 magnification. **(C)** Assessment of the *in vitro* phagocytic uptake of particles by human macrophages. The upper panel shows bright-field and merged images of the unprimed control at 1, 6, and 24 h. The lower panel shows bright-field and merged images of the primed control at 1, 6, and 24 h at ×100 magnification. Data are representative of three independent biological experiments (*n* = 3).

### Priming enhances *in vivo* and *in vitro* phagocytic uptake within macrophages

Evaluation of the priming effect on phagocytic uptake demonstrated that unprimed macrophages exhibited significantly lower phagocytic activity than primed cells. The results demonstrate that priming enhances the ability of macrophages to recognize and engulf microbes and particles effectively. Moreover, a significant increase in the number of phagocytic cells was observed both *in vivo* and *in vitro* between the 6- and 24-h time points, as shown by the red arrows. Particle uptake was higher *in vitro* than *in vivo* from 6 to *24* h. This temporal enhancement indicates that the primed macrophages continued to increase their phagocytic responses over time compared with unprimed cells (Figures 1B,C).

### Priming enhanced intracellular reactive oxygen species generation within *Mycobacterium tuberculosis (H37Rv)-*infected human macrophages

We next examined intracellular reactive oxygen species (ROS) using the DCFH-DA assay. ROS are important components of the macrophage antimicrobial response against intracellular pathogens. *M. tuberculosis* can persist within macrophages by modulating host oxidative responses and interfering with phagosomal and lysosomal antimicrobial mechanisms. In the present study, β-glucan priming in combination with DYDGP particle treatment significantly enhanced intracellular ROS production in *M. tuberculosis*-infected macrophages. At 1 h post-treatment, the increased ROS generation was associated with nicotinamide adenine dinucleotide phosphate oxidase 2 (NOX2) activity, as supported by the inhibitor-based analysis. At 24 h, the sustained ROS response was consistent with a contribution from mitochondrial pathways. Together, these findings indicate that β-glucan priming combined with DYDGP treatment enhances intracellular ROS production and provides supportive evidence for the involvement of both NOX2-associated and mitochondrial ROS pathways at distinct time points. However, these observations suggest pathway involvement rather than provide definitive evidence of the exclusive contribution of these pathways to mycobacterial killing (Figure 2A).

**Figure 2:**
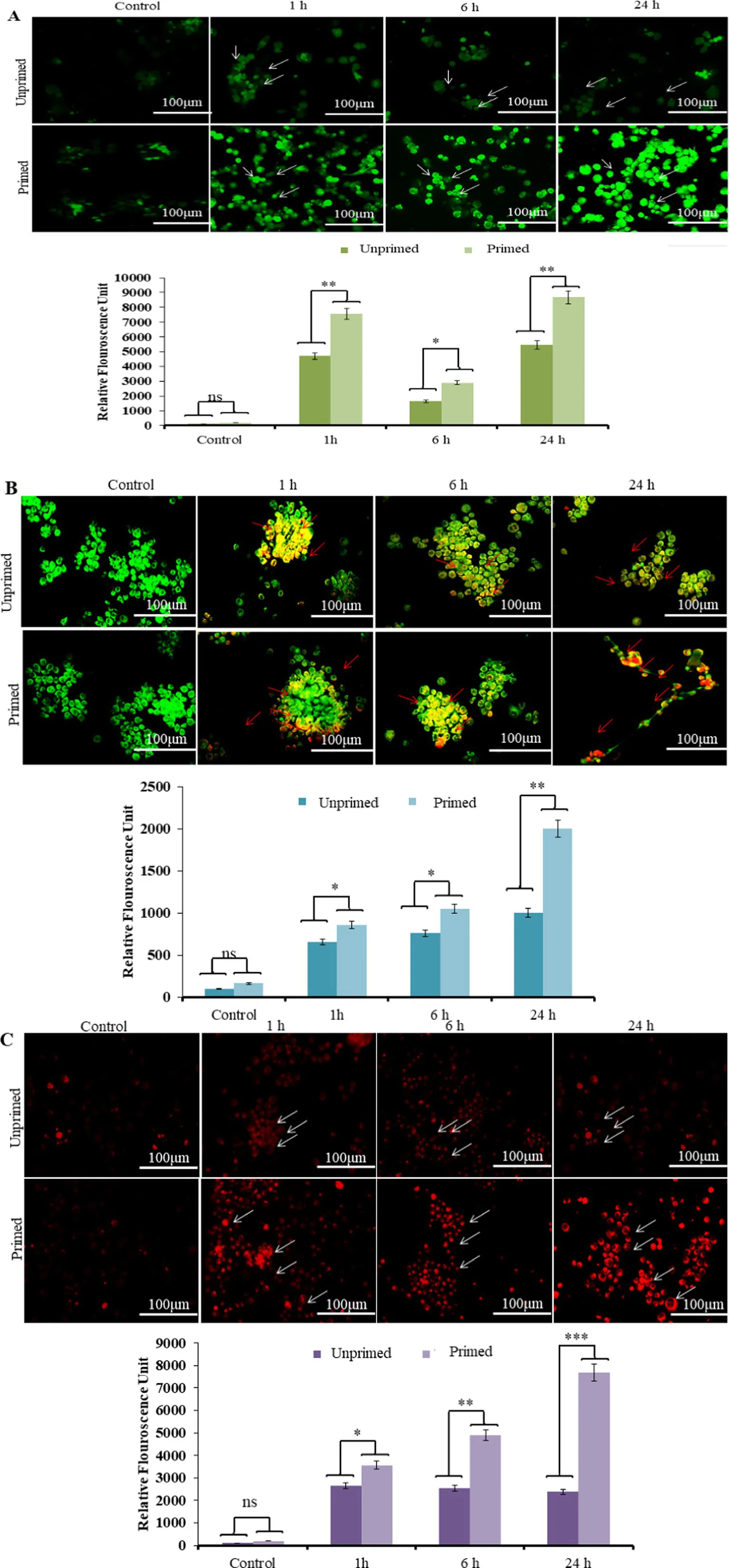
**(A)** Evaluation and quantification of enhanced ROS production by priming and DYDGP particles in *Mycobacterium tuberculosis* (H37Rv)-infected human macrophages. The upper panel shows the unprimed control at 1, 6, and 24 h. The lower panel shows the primed control at 1, 6, and 24 h at ×100 and ×200 magnification. Relative fluorescence intensity was quantified using ImageJ software. **(B)** Evaluation and quantification of acidic vesicular organelle formation in primed *Mycobacterium tuberculosis* (H37Rv)-infected macrophages. The upper panel shows the unprimed control at 1, 6, and 24 h. The lower panel shows the primed control at 1, 6, and 24 h at ×100 magnification. Relative fluorescence intensity was quantified using ImageJ software. **(C)** Evaluation and quantification of phagolysosomal fusion and lysosomal maturation in primed *Mycobacterium tuberculosis* (H37Rv)-infected macrophages. The upper panel shows the unprimed control at 1, 6, and 24 h. The lower panel shows the primed control at 1, 6, and 24 h at ×100 magnification. Relative fluorescence intensity was quantified using ImageJ software. Data are presented as the mean ± SD from three independent biological experiments (*n* = 3), with technical replicates as indicated. *, **, *** denotes the level of statistical significance. It indicates that the corresponding difference is statistically significant at *p* < 0.05.

### Priming enhanced acidic vesicular organelle formation within *Mycobacterium tuberculosis* (H37Rv)-infected macrophages

To further investigate host innate immune response activation and acidic phagosome formation against *M. tuberculosis,* we used AO staining to visualize acidic vesicular organelle (AVO) formation. Our findings revealed that priming and treatment with DYDGP significantly stimulated autophagosome acidification at both 1 and 24 h post-treatment, as shown by the red arrows. The level of acidification at 24 h was much higher in primed cells than in unprimed cells. These findings suggest enhanced acidic vesicular activity in primed macrophages (Figure 2B).

### Priming enhanced phagolysosomal fusion

We evaluated the effect of β-glucan-mediated macrophage priming on phagolysosomal activity using LysoTracker fluorescent dye. Primed and DYDGP-treated macrophages exhibited significantly enhanced phagolysosomal activity, marked by a significant increase in phagolysosomal maturation at both 1 and 24 h post-treatment. Lysosomal maturation was significantly higher at 24 h in primed cells than in unprimed cells, whereas the red arrows show mild activity at 6 h. The sustained phagolysosomal activity observed at both time points demonstrates the potential of DYDGP particles not only to enhance macrophage functionality but also to promote effective mycobacterial clearance. However, these findings suggest this possibility and do not directly establish enhanced bacterial clearance (Figure 2C).

### Priming enhanced host immunity

To further assess the immunotherapeutic efficacy of β-glucan-mediated macrophage priming against H37Rv-infected human macrophages, we conducted a time-dependent analysis of its effects. Our investigation revealed significant immunotherapeutic activity, particularly at the 24-h time point. At this time, priming and DYDGP treatment effectively induced host immunity in primed macrophages, as evidenced by LysoTracker staining. This induction of autophagy-associated lysosomal activation is a critical cellular process that enhances the degradation of intracellular pathogens. Specifically, DYDGP particles facilitated the formation of autophagic vesicles, which then encapsulated and targeted *M. tuberculosis* bacilli for destruction by lysosomal fusion, as evidenced by LysoTracker staining. Inhibition of autophagy-associated lysosomal activation using 3-methyladenine (3-MA) blocked downstream pathways, reducing both vesicular acidification and phagolysosomal fusion in primed macrophages. In contrast, rapamycin was used as a positive control to validate the possible role of autophagy pathways (Figures 3A,B).

**Figure 3:**
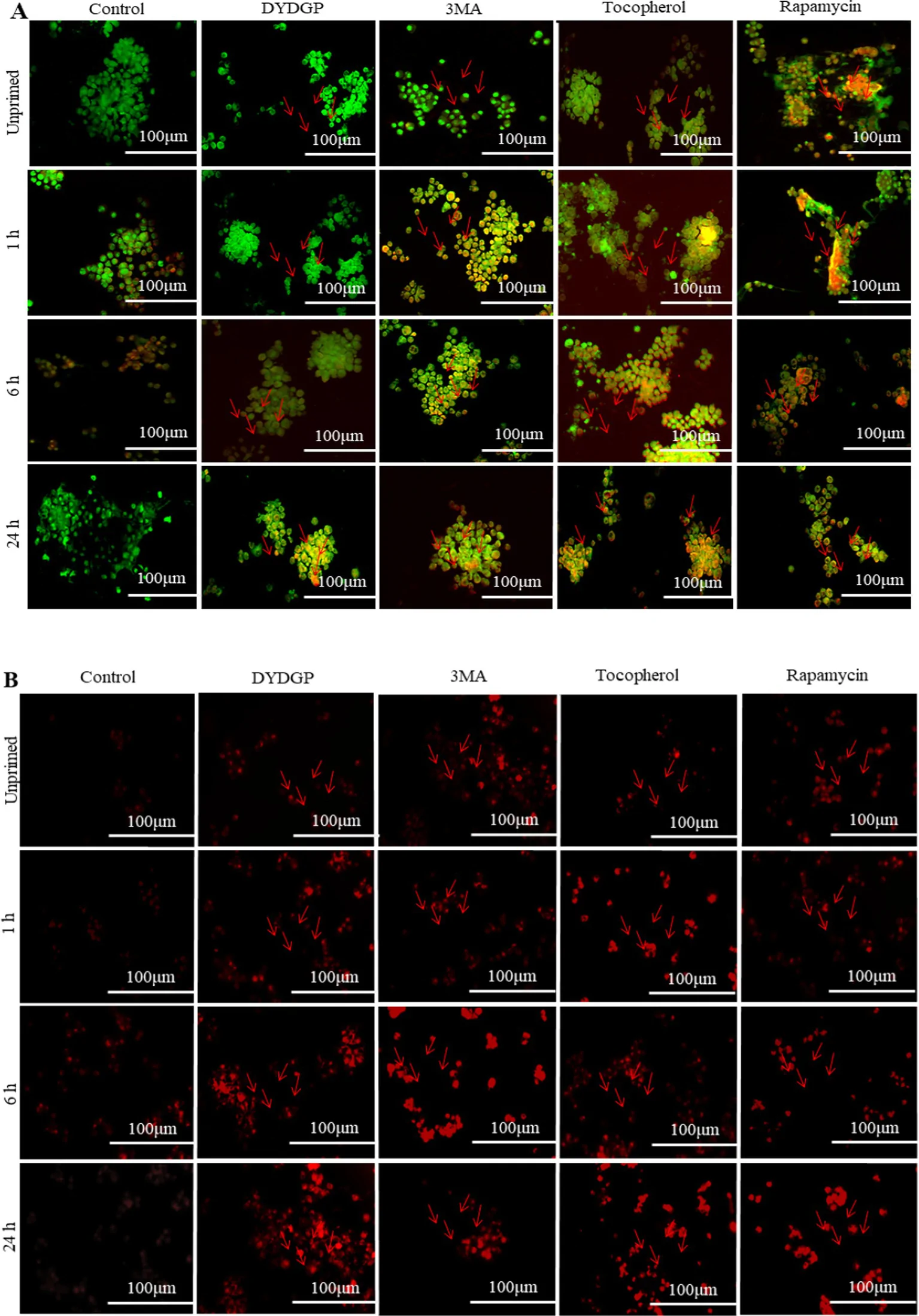
**(A)** Evaluation of acidic vesicular organelle formation in primed *Mycobacterium tuberculosis* (H37Rv)-infected macrophages treated with DYDGP particles. The unprimed panel shows the control at 1, 6, and 24 h; the primed panel shows DYDGP, 3-MA, tocopherol, and rapamycin at ×100 magnification. **(B)** Evaluation and quantification of the effects of DYDGP particles and priming on phagolysosomal fusion and lysosomal maturation in *Mycobacterium tuberculosis* (H37Rv)-infected macrophages. The unprimed panel shows the control at 1, 6, and 24 h; the primed panel shows DYDGP, 3-MA, tocopherol, and rapamycin at ×100 magnification. Data are representative of three independent biological experiments (*n* = 3).

### Priming enhanced intracellular mycobacterial killing

We next examined the antimycobacterial efficacy of β-glucan-mediated macrophage priming using a colony-forming unit (CFU) assay. The CFU results revealed reduced intracellular bacterial loads in primed macrophages in a time-dependent manner. Compared with the unprimed macrophage group, the primed group demonstrated a markedly enhanced antibacterial response over time. In the unprimed condition, the bacterial burden remained relatively high, with log CFU values of 4.2, 4.8, and 5.2 at 1, 6, and 24 h, respectively, indicating progressive bacterial survival and proliferation. In contrast, primed macrophages exhibited substantially lower bacterial counts, with log CFU values decreasing from 3.3 at 1 h to 2.5 at 6 h and further to 2.1 at 24 h, reflecting sustained and time-dependent bacterial clearance (Figure 4). When the primed group was compared with the β-glucan-only and antibiotic-only groups at the corresponding time points, the primed condition consistently showed superior antibacterial efficacy. The β-glucan-only group exhibited log CFU values of 4.2, 4.4, and 4.7 at 1, 6, and 24 h, respectively, suggesting that β-glucan stimulation alone produced only a modest reduction in bacterial burden without inducing robust bactericidal activity. Similarly, the drug group showed log CFU values of 4.2, 4.1, and 3.9, indicating partial bacterial suppression but comparatively weaker clearance than the primed macrophage condition. Importantly, although both the β-glucan and drug groups displayed some inhibitory effects on bacterial growth, their antibacterial activities remained largely comparable across the examined time points. In contrast, the primed macrophage group demonstrated a significantly stronger and more sustained reduction in bacterial load, emphasizing the enhanced antimicrobial potential associated with macrophage priming. These findings suggest that the primed state induces a more effective host-directed antibacterial response than either β-glucan exposure or antibiotic treatment alone.

**Figure 4:**
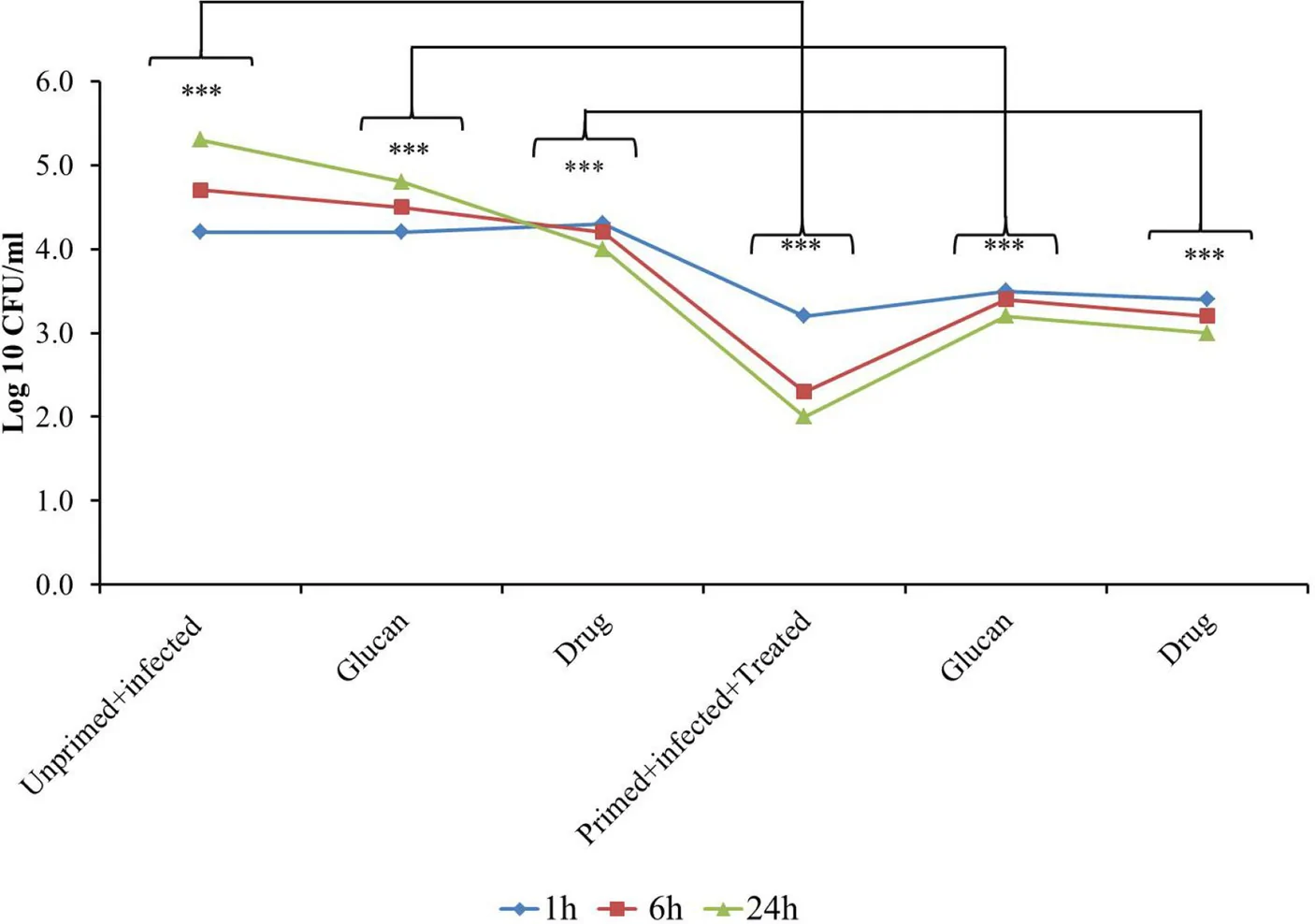
Assessment of intracellular mycobacterial killing following β-glucan priming and DYDGP treatment in *Mycobacterium tuberculosis*-infected human macrophages at different time points. Unprimed, β-glucan-only, antibiotic-only, and β-glucan-primed/DYDGP-treated macrophages were compared at 1, 6, and 24 h post-infection. Data are presented as the mean ± SD from three independent biological experiments (*n* = 3), with technical replicates as indicated. Statistical comparisons among multiple groups were performed using one-way ANOVA followed by Dunnett’s multiple-comparison *post hoc* test, with the designated control group as the reference. *p* ≤ 0.05 was considered statistically significant. *** denotes the level of statistical significance. It indicates that the corresponding difference is statistically significant at *p* < 0.001.

## Discussion

Macrophages play a pivotal role in host defense against *M. tuberculosis* by recognizing, engulfing, and eliminating invading pathogens (Koeken et al., 2019; Rizzetto et al., 2016; Bekkering et al., 2016). In the present study, we investigated whether β-glucan-mediated macrophage priming in combination with DYDGP particle treatment could enhance macrophage antimicrobial responses. Our findings demonstrate that β-glucan priming was associated with enhanced particle uptake, intracellular ROS production, and phagolysosomal activity in *M. tuberculosis*-infected macrophages. These observations support the potential of β-glucan-based approaches as a host-directed strategy against tuberculosis (Hetland et al., 1998; Hetland and Sandven, 2002). However, the findings should be interpreted within the limitations of the current experimental design.

Previous studies have demonstrated that β-glucans can modulate innate immune responses through pattern-recognition receptors and enhance macrophage antimicrobial functions (Soto et al., 2012; Lawlor et al., 2016; Kaufmann et al., 2018; Gutierrez et al., 2004; Soto et al., 2010). Consistent with these observations, our results showed enhanced phagocytic uptake in β-glucan-primed macrophages, particularly at 24 h. Our previous studies also demonstrated that β-glucan-containing particles can stimulate macrophage immune responses and promote intracellular antimicrobial mechanisms. These findings suggest that β-glucan priming may enhance macrophage functional activity; however, the present study does not establish durable primed immunity, and therefore, the observed effects are more appropriately interpreted as β-glucan-mediated macrophage priming (Braian et al., 2023; Kaufmann et al., 2018; Gutierrez et al., 2004; Ferluga et al., 2020).

The enhanced antimicrobial activity observed following combined β-glucan priming and DYDGP treatment may involve both immunomodulatory and drug-delivery effects. DYDGP particles contain a β-glucan-based component capable of interacting with macrophages, while rifabutin provides direct antimycobacterial activity. Previous work has shown that β-glucan-based microparticles can function as immunomodulatory drug-delivery systems (Kumar et al., 2025; Soto et al., 2010, 2012; Lawlor et al., 2016; Kaufmann et al., 2018; Gutierrez et al., 2004). Therefore, the increased antimicrobial activity observed in the combined treatment group may reflemct enhanced macrophage activation, improved intracellular delivery of rifabutin, or a combination of these effects. Because the current experimental design does not completely separate these contributions, further comparative studies using β-glucan alone, unloaded DYDGP, free rifabutin, and rifabutin-loaded DYDGP are warranted.

ROS are important components of macrophage antimicrobial responses, and *M. tuberculosis* has evolved multiple mechanisms to modulate oxidative and phagolysosomal responses during intracellular infection (Braian et al., 2023; Ferluga et al., 2020). In our study, β-glucan priming combined with DYDGP treatment significantly increased intracellular ROS production. The early ROS response was associated with NOX2 activity, whereas the later response was consistent with a contribution from mitochondrial pathways. Our previous findings also suggested a role for NOX2-associated ROS generation following exposure to β-glucan-based particles (Braian et al., 2023; Kaufmann et al., 2018; Gutierrez et al., 2004; Ferluga et al., 2020). Thus, the present observations provide supportive evidence for the involvement of NOX2-associated and mitochondrial ROS pathways at different time points. However, because pharmacological inhibitors such as DPI and rotenone may have limited specificity, these findings should be considered suggestive rather than definitive evidence of pathway-specific ROS generation.

We further observed increased acidic vesicular activity and lysosomal maturation in β-glucan-primed and DYDGP-treated macrophages. These findings are consistent with enhanced phagolysosomal activity and may contribute to improved intracellular antimicrobial responses. Previous studies have linked β-glucan-mediated macrophage activation with autophagy- and lysosome-associated antimicrobial mechanisms (Braian et al., 2023; Horneck Johnston et al., 2024; Gutierrez et al., 2004; Ferluga et al., 2020). Nevertheless, acridine orange and LysoTracker staining primarily provide evidence of acidic vesicular compartments and do not independently establish autophagic flux or phagolysosomal fusion. Similarly, inhibitor-based experiments provide supportive evidence for pathway involvement but do not definitively establish the underlying mechanism. Therefore, the present findings should be interpreted as being consistent with enhanced phagolysosomal and autophagy-associated activity rather than as definitive evidence of increased autophagic flux. The reduction in intracellular mycobacterial burden observed following β-glucan priming and DYDGP treatment provides supportive evidence that this approach can enhance macrophage-mediated control of *M. tuberculosis*. Previous studies have reported that β-glucan-mediated innate immune modulation can improve macrophage antimicrobial activity and influence host responses to mycobacterial infection (Ahmad et al., 2024a,b,c; Braian et al., 2023; Kaufmann et al., 2018; Buffen et al., 2014; Gutierrez et al., 2004; Ferluga et al., 2020). However, because DYDGP simultaneously provides an immunomodulatory β-glucan component and a rifabutin-delivery platform, the relative contributions of host immune activation and enhanced antibiotic delivery cannot be definitively determined from the present experiments. This study has several limitations, including the use of PMA-differentiated THP-1 macrophages and the absence of direct *in vivo* therapeutic evaluation against *M. tuberculosis*. The ROS, acidic vesicular organelle (AVO), and phagolysosomal assays provide supportive rather than definitive mechanistic evidence. In particular, the ROS findings are based on pharmacological inhibition and were not supported by concentration-gradient/dose–response analyses or NOX2 gene-silencing validation; therefore, the involvement of NADPH oxidase and mitochondrial redox pathways should be interpreted cautiously. In addition, the relative contributions of β-glucan priming and rifabutin delivery to the observed antimicrobial effects remain to be fully established. Future studies should validate these findings in primary human macrophages and appropriate *in vivo M. tuberculosis* infection models. Such studies should evaluate bacterial burden, host immune responses, pulmonary pathology, and therapeutic efficacy while incorporating complementary mechanistic analyses, including autophagic flux, phagolysosomal maturation, NOX2-specific genetic validation, and molecular pathways associated with macrophage antimicrobial activity. These investigations will help establish the broader biological relevance and therapeutic potential of the proposed β-glucan-based strategy (see Figure 5).

**Figure 5:**
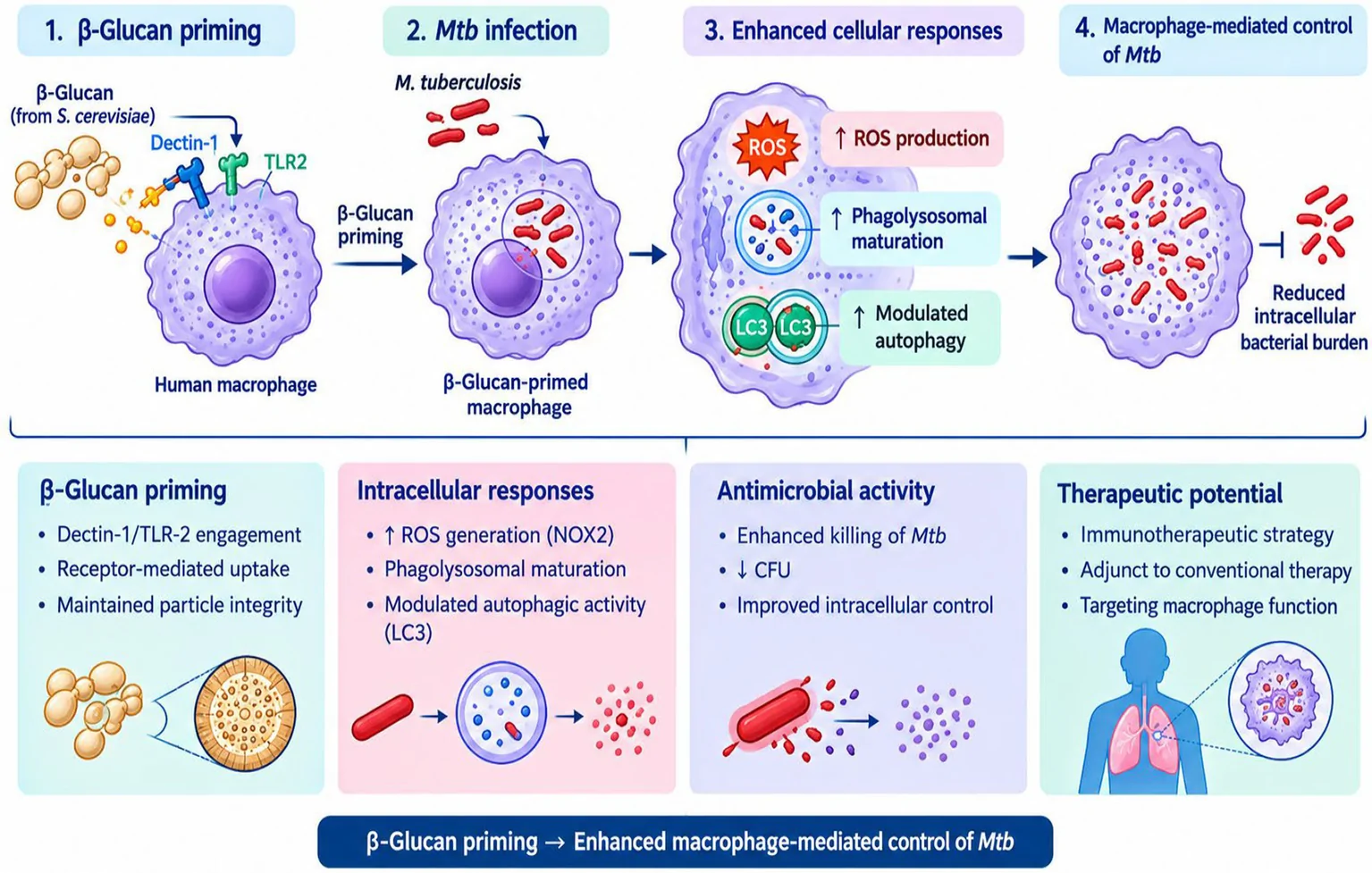
Immunotherapeutic potential of β-glucans in promoting macrophage-mediated control of *M. tuberculosis* infection in human macrophages.

## Conclusion

Our findings suggest that β-glucan priming combined with DYDGP treatment is associated with enhanced macrophage phagocytic, oxidative, and phagolysosomal responses and improved intracellular control of *M. tuberculosis*. These results provide supportive evidence for the potential of β-glucan-based DYDGP as an adjunctive host-directed strategy for tuberculosis. Further studies are required to validate the underlying mechanisms and therapeutic efficacy in appropriate *in vivo* models.

## Data Availability

The datasets presented in this article are not readily available because data made available on request from author. Requests to access the datasets should be directed to Rolee Sharma, roleesharma@csjmu.ac.in.

## Supplementary material

SUPPLEMENTARY Data S1 Final Images of **(1A)** blank YDGP β-glucan particles and **(1B)** drug-loaded DYDGP inhalable microparticles. Particle size distribution was determined using a Malvern Mastersizer. **(2A)** Blank YDGP β-glucan particles and **(2B)** drug-loaded DYDGP Inhalable microparticles. Scanning electron microscopy (SEM) images of blank YDGP β-glucan particles **(3A)** and drug-loaded DYDGP particles **(3B)** at different magnifications. Fourier-transform infrared (FTIR) spectra: (4A) rifabutin, (4B) YDGP β-glucan particles, and (4C) drug-loaded DYDGP microparticles.
